# The Prevalence and Genetic Characteristics of Porcine Circovirus Type 2 in Shandong Province, China, 2018–2020

**DOI:** 10.3390/cimb46120809

**Published:** 2024-11-26

**Authors:** Yuzhong Zhao, Xinyu Cui, Haotian Sang, Shaodong Wen, Lebin Han, Pingping Yang, Yihong Xiao, Yanmeng Hou

**Affiliations:** 1 College of Animal Science and Veterinary Medicine, Shandong Agricultural University, 61 Daizong Street, Tai’an 271018, China; zyz3578@163.com (Y.Z.); 2022110376@sdau.edu.cn (X.C.); 18853851797@163.com (H.S.); 18654553158@163.com (S.W.); hanlebin867@163.com (L.H.); ppyang@sdau.edu.cn (P.Y.); xiaoyihong01@163.com (Y.X.); 2 Shandong Provincial Key Laboratory of Animal Biotechnology and Disease Control and Prevention, Shandong Agricultural University, Tai’an 271018, China

**Keywords:** porcine circovirus, infection, PCV2b, PCV2d, genetic diversity

## Abstract

Porcine circovirus type 2 (PCV2) is an important swine pathogen that has caused considerable economic losses in the global swine industry. During our surveillance of pigs in Shandong, China, from 2018 to 2020, we found that the PCV2 infection rate was 7.89% (86/1090). In addition, we found frequent mixed infections of PCV2 with porcine reproductive and respiratory syndrome virus (PRRSV), classical swine fever virus (CSFV), and porcine herpesvirus (PRV). Thirteen positive clinical samples were selected to amplify the complete genome of PCV2, and were sequenced. Among the 13, we detected two genotypes: PCV2b (1/13) and PCV2d (12/13). This suggests that PCV2d is the dominant genotype circulating in Shandong Province. Additionally, we found three positively selected sites in the ORF2 region, located on the previously reported antigenic epitopes. This investigation will contribute to understanding of the molecular epidemiology and genetic diversity of PCV2 strains in China.

## 1. Introduction

PCV2 is a single-stranded circular DNA virus belonging to the *Circoviridae* family [1,2,3]. Since its identification in the 1990s, PCV2 has been recognized as a primary causative agent of porcine circovirus-associated disease (PCVAD), which includes porcine respiratory disease complex (PRDC), with reproductive failure, porcine dermatitis and nephropathy syndrome (PDNS), congenital tremors (CT), granulomatous enteritis, proliferative and necrotizing pneumonia (PNP), and necrotizing lymphadenitis [4,5]. The immunosuppressive properties of PCV2 increase the susceptibility of infected pigs to secondary infections, contributing to the complex nature of PCVAD and making PCV2 a major challenge for swine health management [6,7].

PCV2 exhibits substantial genetic diversity, with multiple genotypes identified through comprehensive phylogenetic analyses. Among the major genotypes, PCV2a, PCV2b, and PCV2d are widely distributed across global pig populations, with PCV2d recently emerging as the dominant genotype [8,9,10,11]. In addition to these, newer genotypes, including PCV2f, PCV2g, and PCV2h, have been reported, highlighting the virus’s adaptability and genetic variability [12]. This expanding genotypic diversity raises concerns about vaccine efficacy, as existing vaccines may not fully protect against all PCV2 strains, especially the recently emerged variants.

The PCV2 genome, which spans approximately 1766–1768 nucleotides, contains multiple open reading frames (ORFs) that are essential to its replication and pathogenicity [13,14]. In particular, ORF2s are commonly used for PCV2 phylogenetic, epidemiological, and genetic diversity analysis due to their remarkable genetic diversity [15]. Variability within the ORF2 sequence is a primary factor in immune evasion and genotype differentiation, making it a critical focus in studies of PCV2 evolution and pathogenicity. Given the continuous evolution of PCV2, understanding the genetic characteristics of circulating strains is essential for developing effective control measures.

PCV2 has circulated extensively in China since its initial detection, with distinct genotypic shifts observed over time. Originally dominated by PCV2a, the viral population shifted to PCV2b in the late 2000s and subsequently to PCV2d, which now predominates in most regions [9,10,16]. This study aimed to investigate the prevalence and genetic diversity of PCV2 in Shandong Province, a key pig-producing region in China, from 2018 to 2020. By analyzing the genome sequences of PCV2 isolates, we sought to assess the virus’s evolutionary patterns and the selection pressures influencing its persistence, providing valuable insights for vaccine design and the development of control strategies.

## 2. Materials and Methods

### 2.1. Sample Collection and Treatment

A total of 1090 porcine clinical tissue samples, including lung, kidney, spleen, brain, and lymph nodes, were collected from pigs of different sizes in Shandong Province from January 2018 to February 2020. The tissue samples were rapidly transported to the laboratory at 4 °C. They were suspended in sterile phosphate-buffered saline (PBS), and homogenized and centrifuged at 5000 rpm for 3 min. Then, the supernatant was divided into two parts and stored at −80 °C until further testing. Additionally, lung, kidney, spleen, and brain tissues were fixed in 4% formalin for histopathological examination. The tissue samples fixed in formalin and embedded in paraffin are typically sectioned into 4 μm thick slices, followed by staining with hematoxylin and eosin (H&E), and subsequent observation and analysis under a microscope.

### 2.2. DNA Extraction and Sequencing

Total viral DNA was extracted from tissue sample homogenate using commercial kits (Takara Biomedical Technology Co., Beijing, China; catalog number 9766), according to the manufacturer’s instructions. The primers used for amplifying PRRSV, CSFV, and PRV fragments are listed in references [17,18]. The PCR cycling conditions used were 5 min at 95 °C for initial denaturation, 30 cycles of 30 s at 95 °C, 30 s at 58 °C, and 30 s at 72 °C. PCR products were visualized on 1% agarose gels stained with GelGreen. PCR products were recovered by agarose gels, ligated with pMD 18-T vector (Takara Biomedical Technology Co., Beijing, China; catalog number 6011), transformed into DH5α competent cells (Takara Biomedical Technology Co., Beijing, China; catalog number 9057), and cultured for enrichment. Positive clones were sent to the lab for sequencing (Sangon Biotech Co., Shanghai, China).

### 2.3. Phylogenetic Tree Construction

Phylogenetic analyses were conducted using MEGA 7 software (Version number 7.0, Mega Limited, Auckland, New Zealand), employing the maximum likelihood (ML) method to construct the phylogenetic tree. The robustness of the tree topology was evaluated using bootstrap analysis with 1000 replicates to ensure statistical reliability. Fourteen PCV2 sequences with well-defined genotypes, which were downloaded from the GenBank database [19,20,21,22,23,24,25], were included in the analysis to provide comprehensive genotype classification.

### 2.4. Analysis of Viral Sequencing

The nucleotide sequences were edited using the SeqMan module of the DNAStar software package (version 8; DNASTAR, Madison, WI, USA). Genetic similarity calculations were performed using the MegAlign tool within the DNAStar package (version 8; DNASTAR, Madison, WI, USA). The amino acid sequence substitutions of the 13 new PCV2 isolates and the 4 representative PCV2 strains, including AY916791 (PCV2b), AY847748 (PCV2b), HM038031 (PCV2d), and JX535296 (PCV2d), were calculated using MegAlign (DNAStar, Madison, WI, USA) and visualized using EPS Print 3.0 [26].

### 2.5. Selection Pressure Analysis of ORF2

The selection pressure on ORF2 coding sequences was assessed using the Datamonkey server (http://www.datamonkey.org), which analyzes the dN/dS ratio to detect positive selection [27]. This analysis employed methods including Single-Likelihood Ancestor Counting (SLAC), Fixed-Effects Likelihood (FEL), Mixed-Effects Model of Evolution (MEME), and Fast Unconstrained Bayesian Approximation (FUBAR), all of which evaluate site-specific selection pressures. Positive selection sites were considered significant if two or more methods indicated positive selection (*p* < 0.1 for SLAC, FEL, MEME, or posterior probability > 0.9 for FUBAR), suggesting their potential involvement in immune escape mechanisms.

## 3. Results

### 3.1. Clinical Signs and Gross Lesions

**Figure 1:**
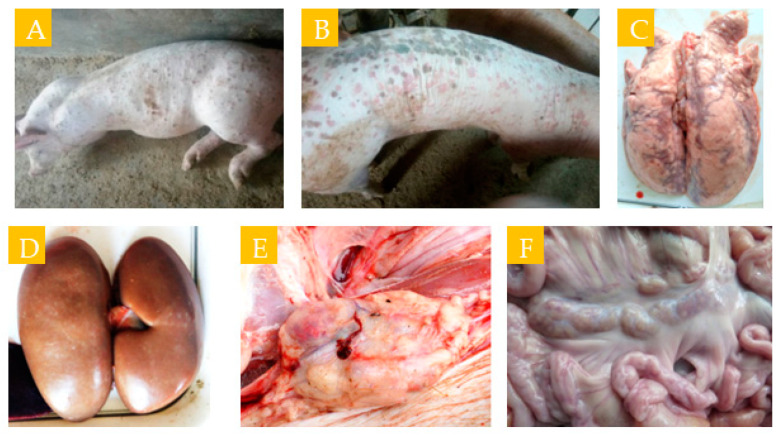
Clinical signs and gross lesions observed in pigs. (**A**,**B**) Red spots on skin surface; (**C**) gray–white spots on the kidney surface; (**D**) focal solid lesions on the lung surface; (**E**) gray–white lymph node enlargement in the groin; (**F**) gray–white lymph node enlargement in the mesentery.

PCV2-infected pigs showed mainly lethargy, dyspnea, diarrhea, pale and jaundiced skin, some with red or brown lesions on the skin (Figure 1A,B), and enlarged lymph nodes on the body surface. Clinical autopsy usually shows focal or diffuse solid lesions in the lungs (Figure 1C), gray–white spots on the kidney surface (Figure 1D), and enlarged gray–white inguinal and mesenteric lymph nodes (Figure 1E,F).

### 3.2. Histopathological Changes

**Figure 2:**
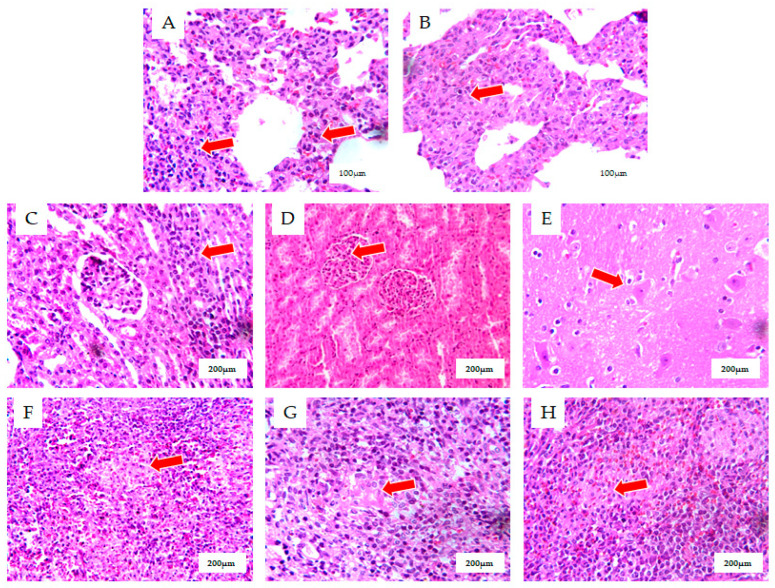
(**A**,**B**), Lungs with significant thickening of alveolar walls, macrophage and lymphocyte infiltration, and hyperplasia presenting as interstitial pneumonitis lesions (HE X 400). (**C**,**D**) Kidneys with the focal infiltration of macrophages and lymphocytes between renal tubules and glomerular macrophage infiltration, and hyperplasia presenting as acute interstitial nephritis (HE X 200). (**E**) Brain with peri-neuronal gliosis and small perivascular lymphocytic infiltration (HE X 200). (**F**,**G**) Lymph nodes with the absence of lymphocytes and macrophage hyperplasia (HE X 200). (**H**) The absence of lymphoid fines in the spleen with an unremarkable white medullary structure and macrophage hyperplasia (HE X 200). The lung, kidney, brain, and spleen tissues were fixed in formalin, embedded in paraffin, stained with hematoxylin and eosin, and examined under a microscope.

In the lungs, there was a significant thickening of the alveolar walls with infiltration and proliferation of macrophages and lymphocytes, showing interstitial pneumonia lesions (Figure 2A,B). In the kidneys, there was focal infiltration of macrophages and lymphocytes between renal tubules, and the infiltration and proliferation of glomerular macrophages, showing acute interstitial nephritis (Figure 2C,D). In the brain, there was perineuronal glial cell hyperplasia and a small perivascular lymphocytic infiltrate (Figure 2E). Lymphocytes were absent and macrophages proliferated in the lymph nodes (Figure 2F,G). Lymphoid fines were absent in the spleen, white medullary structures were not evident, and macrophages were proliferating (Figure 2H).

### 3.3. Pathogens Detected in Clinical Samples

**Figure 3:**
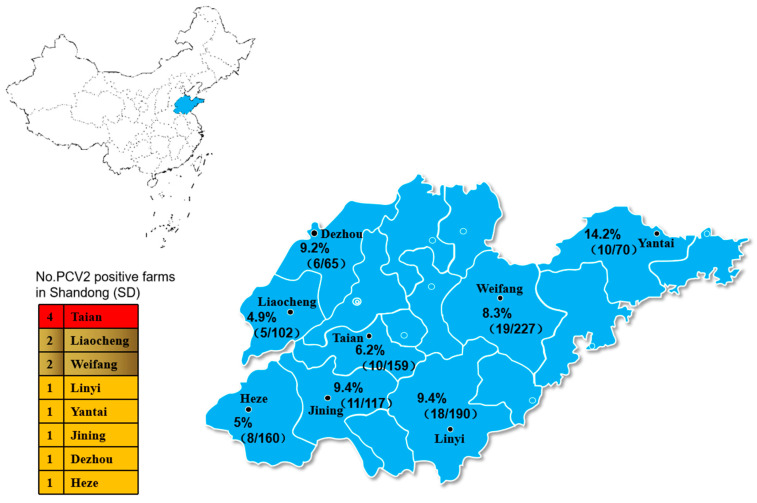
Results of PCV2 pathogenicity testing in different municipalities in Shandong, 2018–2020. The number of PCV2-positive pig farms participating in the study in each city was as follows: Taian (4), Liaocheng (2), Weifang (2), Linyi (1), Yantai (1), Jining (1), Dezhou (1), and Heze (1). The positivity rates of PCV2 clinical samples participating in the study in each city were as follows: Tai’an (6.2%), Liaocheng (4.9%), Weifang (8.3%), Linyi (9.4%), Yantai (14.2%), Jining (9.4%), Dezhou (9.2%), and Heze (5%).

Pathogenic testing was performed on 1090 tissue samples from pig farms in Shandong Province using PCR assays. Of these 1090 samples, we found that 86 (7.8%) tested positive for a PCV2 infection. PCV2 infection occurred in all eight prefectures tested (Figure 3). The PCV2 positivity rate was 9.8% (39/396) in 2018 and 6.7% (47/694) in 2019 (Figure 3). Thirteen PCV2s were sequenced to further analyze the PCV2 characteristics.

### 3.4. Co-Infection Rate

The samples testing positive for PCV2 by PCR were also tested for co-infection status with PRRSV, CSFV, and PRV. Of the PCV2-positive samples, 54.65% (47/86) were co-infected with other porcine pathogens (Table 1). Co-infection with PRRSV was the most common (37.20%), followed by CSFV (24.42%) and PRV (10.47%) (Table 1). Infections with PRRSV and CSFV were detected in all eight prefectures and PRV in six. Among the dual infections, PCV2 had the highest co-infection rate with PRRSV (42.55%), followed by CSFV (19.15%) and PRV (4.26%) (Table 1). Among the triple infections, PCV2 had the highest co-infection rate with PRRSV + CSFV (19.15%), followed by CSFV + PRV (8.51%) and PRRSV + PRV (6.38%) (Table 1). Five types of co-infection were detected in Tai’an; four types of co-infection in Weifang; three types of co-infection in Linyi, Yantai, Jining and Heze; two types of co-infection in Liaocheng; and one type of co-infection in Dezhou (Table 1).

### 3.5. Phylogenetic Analysis

**Figure 4:**
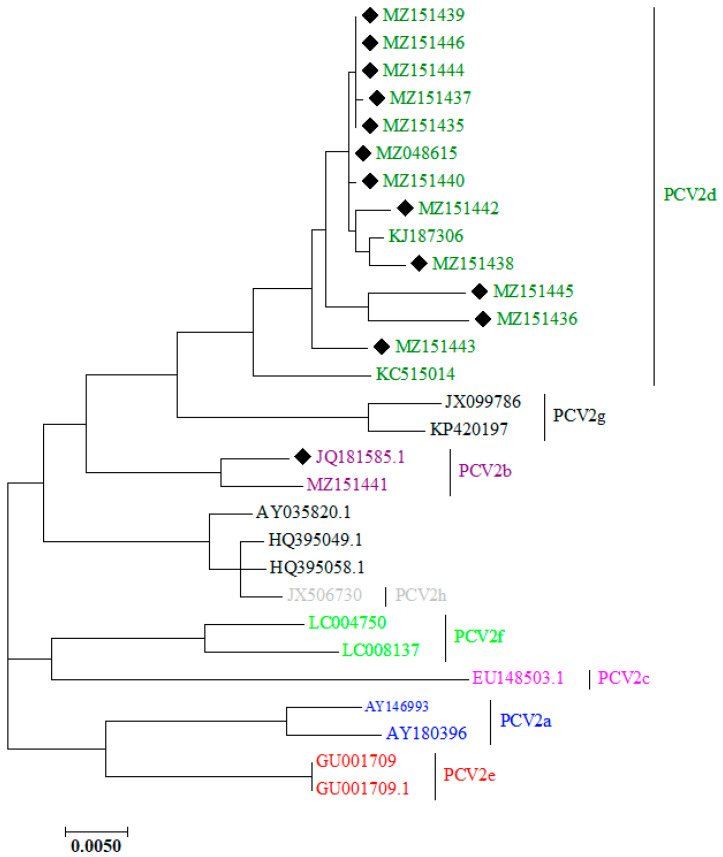
Phylogenetic tree based on ORF2. Gene sequences of the 13 new PCV2s and 14 other representative strains constructed with the MEGA 7 software using the maximum likelihood (ML) method. Bootstrap replications (1000) were employed to assess the reliability of the tree. Black diamonds indicate the 13 new PCV2s in this study.

The genome size of the 13 new PCV2s was 1767 nucleotides in length. The ORF2 gene of the 13 new PCV2s shared 94.6% homology. The ORF2 gene of the 13 new PCV2s and 14 reference PCV2s shared 80.6–100% homology. All strains shared 90.1–100% nucleotide sequence homology for the whole genome. Comparing the 13 PCV2s with the corresponding sequences of the highest-nucleotide-homology strains in GenBank, the nucleotide sequence homology between them was 99.1–100% (Table 2). To better understand the genetic evolution of PCV2, we constructed a phylogenetic tree of the sequenced ORF2 genes of the 13 new PCV2s together with 14 reference sequences with well-defined genotypes. The phylogenetic tree revealed that PCV2 clustered into five genotypes: PCV2a, PCV2b, PCV2c, PCV2d, and PCV2e (Figure 4). The 13 new PCV2s belonged to the PCV2b (1/13) and the PCV2d (12/13) genotypes.

### 3.6. Amino Acid Analysis of ORF2

**Figure 5:**
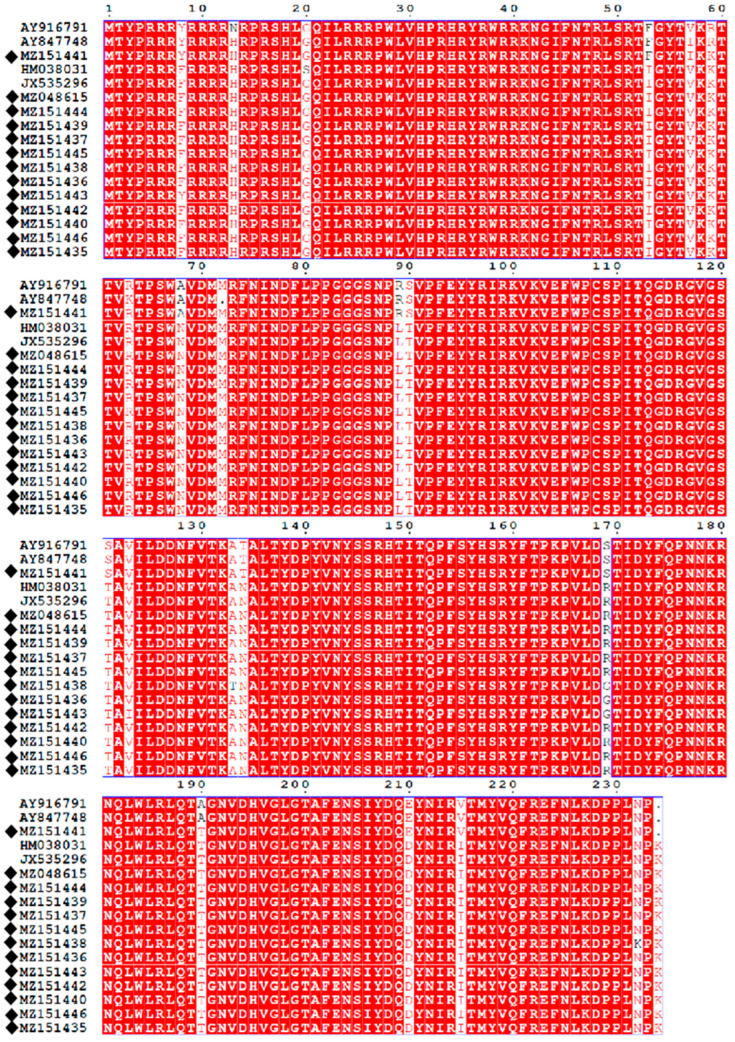
Alignment of ORF2-coding sequences of the 13 new PCV2s and 4 PCV2 reference strains. The diagram was plotted by EPSprint 3.0. Black diamonds indicate the 13 new PCV2 in this study.

To assess the variation in ORF2 coding sequences, the ORF2 coding sequences of 17 PCV2s (including 13 new PCVs and 4 reference strains) were aligned. Analysis of the typical motifs of ORF2 (86–91 and 190/191/206/210) for both genotypes (PCV2b and PCV2d) revealed that the amino acid residues at positions 86–91 were SNPRSV in the PCV2b strain and SNPLTV in the PCV2d strain, while the amino acid residues at positions 190/191/206/210 were A(T)GIE in the PCV2b strain and TGID in the PCV2d strain. Furthermore, compared with the PCV2b strain, the PCV2d strain harbored an extra lysine residue at the C-terminal end at position 234. In the study, compared with the PCV2b/PCV2d strain, the MZ151438 strain contained two unique amino acids (133 K, 232 K) in the ORF2-coding sequences. In addition, the MZ151438 strain contained a unique amino acid (123I) in the ORF2-coding sequence (Figure 5).

### 3.7. Selection Pressure Analysis

The selection pressure on the ORF2-coding sequences of the 13 new PCVs and the 42 reference PCV2 strains (Table 3) was analyzed. Positive selection was found in positions 190 and 191 of the ORF2-coding sequence by the FEL, FUBAR, and MEME methods (*p* < 0.1), while positive selection was found in positions 63 by the FEL and FUBAR methods (*p* < 0.1) (Table 4). 

## 4. Discussion

Since its discovery in 1996, PCV2 has undergone significant genetic and epidemiological changes in pig populations [4,28], leading to diverse clinical outcomes and challenges in controlling its prevalence. Despite extensive vaccination efforts, PCV2-associated diseases, such as PCVAD, continue to pose a major threat to the global swine industry. In this study, we investigated the genetic characteristics, co-infection rates, and phylogenetic relationships of PCV2 strains circulating in Shandong Province, China, from 2018 to 2020. Our findings highlight the persistence of PCV2 in pig populations and underscore the complexity of its control, especially in the context of co-infections with other pathogens such as PRRSV, CSFV, and PRV.

Epidemiological data from this study show that PCV2 is widely prevalent in Shandong Province, with a positivity rate of 7.8% in pig tissue samples. The prevalence of PCV2 infection varied by year, with the positivity rate in 2018 (9.8%) higher than that in 2019 (6.7%). The widespread occurrence of PCV2 across all eight counties sampled indicates that the virus remains a significant issue for pig farms in the region. Notably, we found a high co-infection rate of PCV2 with other swine pathogens, particularly PRRSV, CSFV, and PRV. More than 54% of PCV2-positive samples were co-infected with at least one of these pathogens, with PRRSV being the most common co-infecting virus (37.2%). Previous studies have confirmed that PCV2 can suppress the host’s immune response, thereby facilitating co-infection with other pathogens [29]. Co-infection with PCV2 and other viruses has been shown to enhance pathogenicity, provoke stronger cytokine responses, and result in more severe clinical symptoms, lung lesions, and increased mortality [30,31]. Further epidemiological studies have suggested that PCV2-positive pigs exhibit significantly higher morbidity and severity of diseases caused by porcine parvovirus (PPV) and PRV [32]. The high rate of co-infection is concerning as it may exacerbate disease severity, complicate diagnosis, and hinder the effectiveness of vaccination and treatment strategies. The co-infection patterns observed in this study emphasize the need for a comprehensive disease control approach that addresses not only PCV2, but also other prevalent pathogens in the region.

In China, all recognized PCV2 genotypes, with the exception of PCV2c, have been identified since its introduction in 2000 [33,34]. Prior studies have shown that PCV2a and PCV2b were the predominant genotypes circulating in China between 2006 and 2007, with a notable shift from PCV2a to PCV2b occurring between 2009 and 2010, suggesting a decline in the significance of PCV2a and the rise in PCV2b as the dominant genotype [17,35]. Recently, a genotype shift to PCV2d has been observed, with PCV2d emerging as the new dominant genotype in China, while PCV2e was detected for the first time in 2017 [36]. In our study, we found that 92.3% (12/13) of the isolated PCV2 strains were classified as PCV2d, while 7.7% (1/13) belonged to the PCV2b strain, confirming the dominance of PCV2d in the pig farms of Shandong province.

Genetic evolutionary analysis of the ORF2 sequence of PCV2 enabled us to differentiate between various genotypes. Specific motifs within the ORF-coding sequences have been identified across different genotypes, including amino acid residues at positions 86–91, which are associated with the two genotypes (PCV2b and PCV2d) and characterized as SNPRSV/SNPLTV, as well as residues at positions 190, 191, 206, and 210, represented as A(T)GIE and TGID, respectively [34]. Our findings corroborated these previously reported motifs. Notably, a unique lysine residue was identified at position 133 of strain MZ151438, which may play a role in the virulence of PCV2 [37]. The ORF2-encoding sequence is subject to selective pressure and is a key target for the immune response, exhibiting a higher evolutionary rate compared to the remainder of the PCV2 genome [38,39,40]. In our analysis, we identified three positive selection sites (63, 190, 191) in the ORF2 of Chinese PCV2 strains that are under selective pressure, all of which are associated with immune-related epitopes. Variations at these sites may contribute to the immune evasion capabilities of PCV2, facilitating its continued spread within the pig population.

In summary, the study emphasizes the need for continuous epidemiological surveillance and research to understand the dynamics of PCV2 infection and its interactions with other pathogens. This understanding is crucial for developing effective preventive measures and controlling the spread of PCV2 in pig populations.

## 5. Conclusions

In conclusion, our study highlights the widespread prevalence and genetic diversity of PCV2 in Shandong Province, China, emphasizing the persistence of the virus despite ongoing vaccination efforts. The high co-infection rates of PCV2 with other swine pathogens, particularly PRRSV, CSFV, and PRV, underscore the complexity of controlling PCV2-related diseases, which are often aggravated by mixed infections. These findings suggest that effective control strategies should go beyond targeting PCV2 alone and include measures to address the broader spectrum of pathogens circulating in the region. Furthermore, the study underscores the need for the continuous monitoring of PCV2 and its co-infecting agents to better inform vaccine development and improve disease management in the swine industry.

## Figures and Tables

**Table 1:** The co-infection of PCV2-positive samples with other pathogens of interest in pigs.

	Taian	Liaocheng	Weifang	Linyi	Yantai	Jining	Dezhou	Heze	Sum	
PCV2-positive number	10	5	19	18	10	11	6	8	86	
Co-infection positives	8	4	11	7	4	5	3	5	47	
	number of infections								number of infections	co-infection rate
Double infection										
PRRSV	4	3	6	5	0	2	0	0	20	42.55%
CSFV	1	0	3	0	2	1	0	2	9	19.15%
PRV	1	0	0	1	0	0	0	0	2	4.26%
Triple infection										
PRRSV + CSFV	1	1	1	0	1	0	3	2	9	19.15%
PRRSV + PRV	1	0	0	1	0	0	0	1	3	6.38%
CSFV + PRV	0	0	1	0	1	2	0	0	4	8.51%
The total number of co-infections with a certain pathogen										
PRRSV	6	4	7	6	1	2	3	3	32	37.20%
CSFV	2	1	5	0	3	3	3	4	21	24.42%
PRV	2	0	1	2	1	2	0	1	9	10.47%

**Table 2:** The PCV2s in NCBI with the highest nucleotide homology with the 13 new PCV2s.

Strain/GenBank Accession Number	Virus Sequences with the Highest Genetic Similarity	Homology
TA001/MZ048615	KNU/MT814846	100%
JN226/MZ151435	SZ1101/JX406420	100%
LY012/MZ151436	SZ1101/JX406420	100%
TA007/MZ151437	SZ1101/JX406420	99.9%
LC001/MZ151445	Han8/JQ181600	99.9%
LC014/MZ151438	YiY-3-46-2/KU317499	99.7%
TA002/MZ151444	HN1401/KY940536	99.1%
TA005/MZ151439	QZ1410/MG732832	99.8%
HZ025/MZ151446	SZ1101/JX406420	100%
DZ365/MZ151440	KNU/MT814846	99.9%
WF001/MZ151441	SD-QH/KJ511872	99.9%
WF005/MZ151443	GXBB1501211/MH756609	99.8%
YT265/MZ151442	GC161228/KY659553	99.9%

**Table 3:** ORF2 sequence of the PCV2 strains which were obtained from GenBank was used for the analysis of selection pressure in this study.

GenBank Accession Number	Year	GenBank Accession Number	Year	GenBank Accession Number	Year
**MF139056**	1996	EF190922	2004	JN119255	2011
**MF139068**	1997	DQ141322	2005	MF679600	2012
**MF139070**	1998	EF197986	2005	KC821781	2013
**MF139066**	1998	EF028202	2006	MG229675	2013
**MF139067**	1998	EF190927	2006	KY940530	2013
**MF139075**	1998	EF592575	2007	KU311028	2014
**MF139061**	1999	FJ440338	2008	MG229674	2014
**AF381176**	2001	FJ644927	2008	MF679570	2015
**AY035820**	2001	GQ845028	2009	MF142276	2016
**AY181946**	2002	GQ845027	2009	MF679602	2017
**AY291318**	2002	HQ395052	2010	MG813261	2017
**AY510375**	2003	HQ693093	2010	MF679578	2017
**AY579893**	2003	JQ955679	2010	MG798696	2018
**DQ104419**	2004	JF928005	2011	MG786933	2018

**Table 4:** Selection pressure analysis of the OFR2-coding sequences of PCV2s using four analytical methods.

Codon a	FEL		SLAC		FUBAR		MEME	
	dN-dS	*p*-Value	dN-dS	*p*-Value	β-α	Post. Pro b	β+	*p*-Value
63	Infinity	0.089	None	-	5.791	0.976	None	-
190	Infinity	0.024	None	-	7.097	0.994	4.01	0.04
191	Infinity	0.069	None	-	3.493	0.958	3.52	0.07

The positive selection sites found by at least two methods are shown. When the *p*-value was below 0.1 in FEL, SLAC, and MEME, and the posterior probability was above 0.9 in FUBAR, considered under positive selection. β+: the MLE of the unconstrained β non-synonymous rate; a: positively selected sites (AA); b: posterior probability.

## Data Availability

The data that support the findings of this study are included within the article.
